# Hourly Activity and Natural Infection of Sandflies (Diptera: Psychodidae) Captured from the Aphotic Zone of a Cave, Minas Gerais State, Brazil

**DOI:** 10.1371/journal.pone.0052254

**Published:** 2012-12-19

**Authors:** Gustavo Mayr de Lima Carvalho, Reginaldo Peçanha Brazil, Lara Saraiva, Patrícia Flávia Quaresma, Helbert Antônio Botelho, Mariana Campos das Neves Farah Ramos, Ana Paula Lusardo de Almeida Zenóbio, Paula Cavalcante Lamy Serra e Meira, Cristiani de Castilho Sanguinette, José Dilermando Andrade Filho

**Affiliations:** 1 Grupo de Estudos em Leishmanioses, Centro de Pesquisas René Rachou/Portuguese Fundação Oswaldo Cruz, Belo Horizonte, Minas Gerais, Brazil; 2 Departamento de Fisiologia e Bioquímica de Insetos, Instituto Oswaldo Cruz/Portuguese Fundação Oswaldo Cruz, Rio de Janeiro, Rio de Janeiro, Brazil; Swedish University of Agricultural Sciences, Sweden

## Abstract

Sandflies are holometabolous insects that are of great epidemiological importance in the neotropical region as vectors of leishmaniases. Caves are ecotopes that significantly differ from external environments and, among the insects that live or visit their internal area and adjacent environment, sandflies are commonly found. Based on this context, the objective of this work was to examine the period of activity of sandflies in the cave environment in the aphotic zone. Thus, four sandfly captures were conducted, one in each season of the year, in a cave where studies on the bioecological aspects of sandfly fauna have been conducted since 2008. In this same study, we have also noticed the presence of flagellates in some captured females. Catches were carried out for 24 hours using a Shannon trap, light bait, and cave walls were actively searched. We collected a total of 638 sandflies, representing 11 species. The most abundant species and with more intense period of activity were, in descending order: *Lu. cavernicola* (62%), *Ev. spelunca* (16%) and *Ev. sallesi* (14%). A total of 69 females were dissected to check for natural infection, and in five specimens we found living flagellated forms: two *Ev. spelunca*, two *Ev. sallesi* and one *Sc. sordellii*. This study shows that the activity of some species caught in the aphotic zone of the cave, especially *Lu. cavernicola*, differs from what has already been reported in previous sandfly captures, which are almost always conducted at night and during twilight. The existence of sandflies that were naturally infected with flagellates and the lack of awareness regarding the behaviour of sandflies in cave environments are strong indicators of the need for further study on this group of insects in this ecotope, as a safety measure to protect the visitors of such environment.

## Introduction

Leishmaniasis is a complex infectious disease caused by protozoan parasites of the morphologically homologous genus *Leishmania* Ross, 1903 (Kinetoplastida: Trypanosomatidae), all with varying degrees of specificity to invertebrate host [1]. The main form of parasite transmission to humans and other mammalian hosts is through the bite of female flies of the family Psychodidae, subfamily Phlebotominae, popularly known as sandflies. The occurrence of this parasite in a given area depends primarily on the putative presence of the vector and a host/reservoir [2].

Caves are natural cavities, often found in limestone regions, in which the composition favors the dissolution of rocks by acidic water. The cave environment is characterized by small daily temperature changes, which are always similar to the mean temperature of the external environment; the relative humidity tends to range from 95% to 100% [3].

Thus, the caves are considered stable and peculiar environments that are important for the balance of ecosystems, since inside, the microclimate shows little variation of these factors. Several Brazilian caves stand out on the international scene for their size and rarity. Caves are relatively more isolated than others ecosystems, with specific physical and constant environment, thus, their fauna and flora present peculiar aspects. With more than 600 species that have already been classified, the cave fauna of Brazil is the richest in South America [4].

Among the insects that live in or visit the caves and their surrounding environments, the sandflies deserve special attention, not only because several species are vectors of diseases like arboviruses, bartonellosis and leishmaniasis [5], [6], but also because they cause discomfort to visitors, because of their painful bite, often causing allergic reactions.

There is today an important scientific question, and an urgent need to better study the cave environment. Many caves are open to visitors without having gone through any type of scientific examination or study.

The number of sandflies found is increasing rapidly, with descriptions of new species every year, including species associated with caves [7], [8], [9]. However, with regard to biology, there is still a large gap in the understanding of these insects. Sandflies are considered insects with crepuscular and nocturnal habits. Male and female sandflies during the day remain in protected locations with rarely changing environments. However, there are only a few reports on the biological behaviour of sandflies found in caves.

Accordingly, the goal of our work was to analyze the period of activity of sandflies in the cave environment, specifically in the aphotic zone, where there is no light throughout the day, as well as to observe the natural infection of captured females.

## Materials and Methods

### Ethics Statement

Catches were made in a cave situated on a private farm. A term of consent was established to run the captures in this cave.

### Study Area

This study was conducted in the municipality of Lassance, located in northern Minas Gerais state, in the microregion of Pirapora. The city covers an area of 3.204,213 km^2^ and has an estimated population of 6.484 inhabitants [10]. The region has nine caves recorded in the National Register of Caves of Brazil (CNC) [11]. The cave chosen for this study has not yet been cataloged and is situated at about 20 km from the city, located on a private farm and belonging to Speleological Province of Bambuí, which is situated partly in the Southeastern region of Tocantins, central east and Southeast of Goiás, central West and North West of Minas Gerais and west of Bahia. Other studies that address the bioecological aspects of sandflies caught in this cave have been carried out since 2008 (unpublished data). This is a limestone cave with a horizontal extension of approximately 100 meters that is located at an altitude of approximately 700 meters, with the following coordinates: −17°59′40.01″S, −44°39′3.23″W.

### Sampling of sandflies

**Figure 1:**
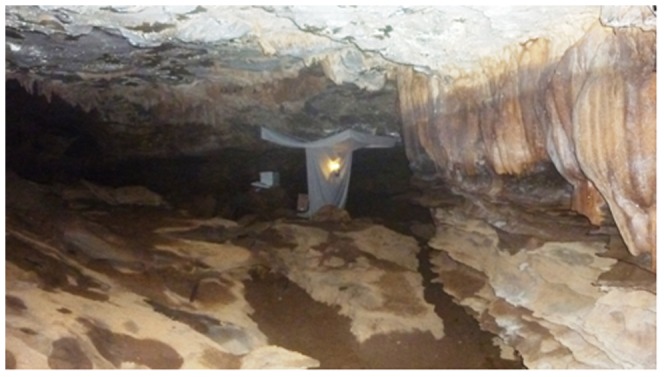
Shannon trap inside the cave, Lassance/MG.

Catches were made with a manual aspirator, at the end of cave (aphotic zone, 60 meters from the entrance), using a Shannon trap [12] (Figure 1), a light bait, and cave walls were actively searched. The samples were standardized as follows: two people conducting the collections, which lasted ten minutes in every hour, over 24 hours. Captures were performed in August 2010 (winter), November 2010 (spring), February 2011 (summer) and May 2011 (autumn). The sandflies were separated into plastic pots, according to their time of capture. The classification used for the identification of sandflies was that proposed by Galati [13].

### Search of natural infection

The females were separated and kept alive in the pots, for us to verify natural infection using the digestive tract dissection technique. Only the living specimens were used for this study.

When flagellated forms were found, a suspension containing PBS 1× with antibiotics (streptomycin and penicillin) and intestinal contents of insects were inoculated in hamsters and in culture medium NNN-Schneider, in an attempt to isolate the parasite.

### Data statistical analysis

The Graph Pad Instat software was used for statistical analyses. Chi-square test df = 5 was used to compare species distribution on day shifts (daylight and darkness) in the overall analysis. Pairwise analyses of species distribution was performed using Fisher's exact test, two sided. Comparison of species distributions across all seasons were performed using the Chi-square test df = 12. Pairwise analysis of species distributions in the seasons was performed using the Chi-square df = 4.

## Results

### Hourly activity

**Figure 2:**
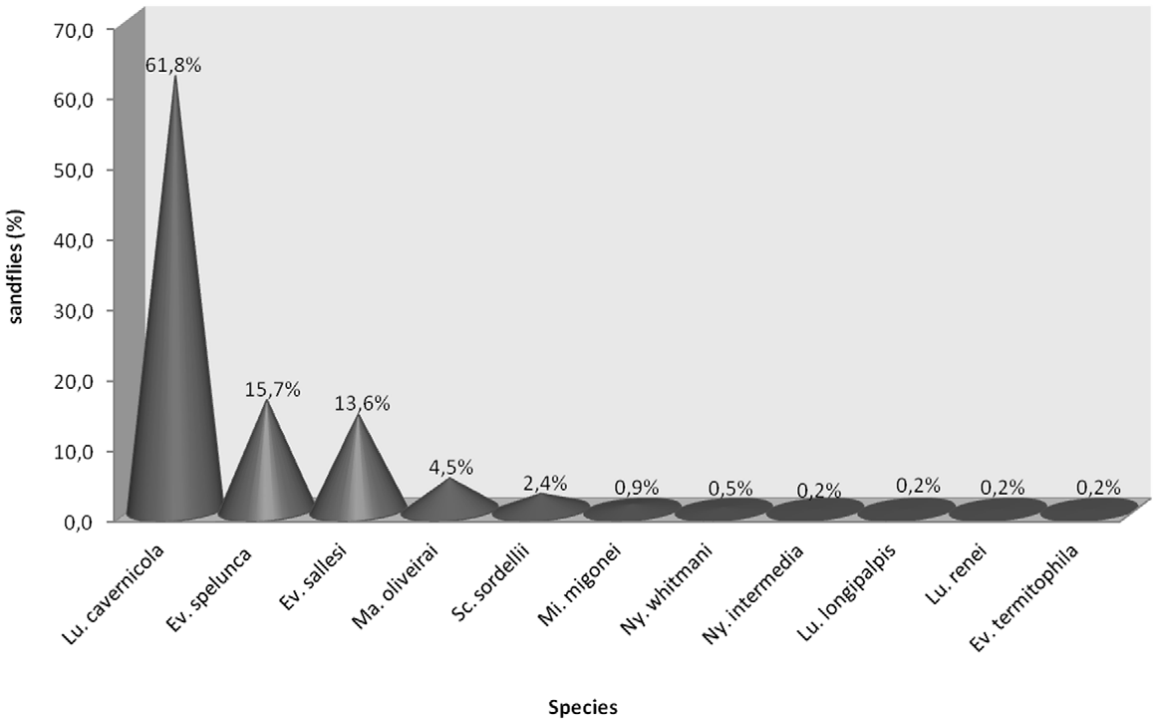
Total of sandflies captured with Shannon trap in winter, spring, summer and autumn, in the aphotic zone of the cave - Lassance/MG – Brazil.

Considering all catches together, winter (August 2010), spring (November 2010), summer (February 2011) and autumn (May 2011), a total of 638 sandflies were collected, 358 (56%) males and 280 (44%) females, representing 11 species, where the most abundant species were, in descending order: *Lutzomyia cavernicola* (Costa Lima, 1932) (62%), *Evandromyia spelunca* Carvalho, Sanguinette, Brazil & Andrade Filho, 2011 (16%) and *Evandromyia sallesi* (Galvão & Coutinho, 1939) (14%) (Figure 2).

**Figure 3:**
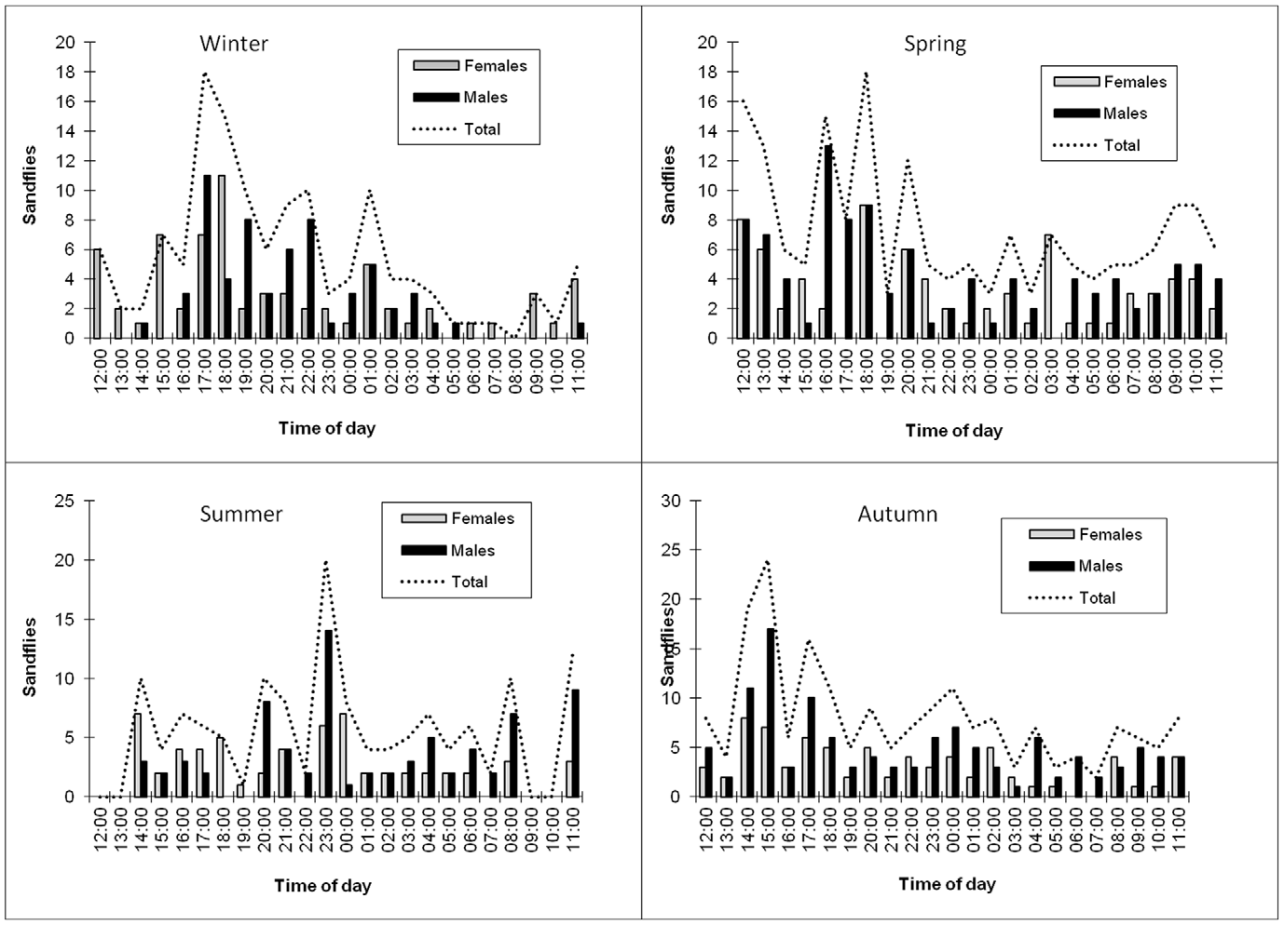
Dispersion of sandflies over the 24 hours, in the four seasons of capture, inside the cave, Lassance/MG.

Figure 3 shows the distribution of species over 24 hours, in the four seasons when captures took place. The following parameters were taken into account: a photoperiod with a light/dark cycle (LD) 12∶12, observed in the external environment, and peak activity of sandflies in all seasons during scotophase (18:00–5:00). However, it has been noted that sandfly activity across all seasons also occurred during periods of photophase (6:00–17:00).

**Figure 4:**
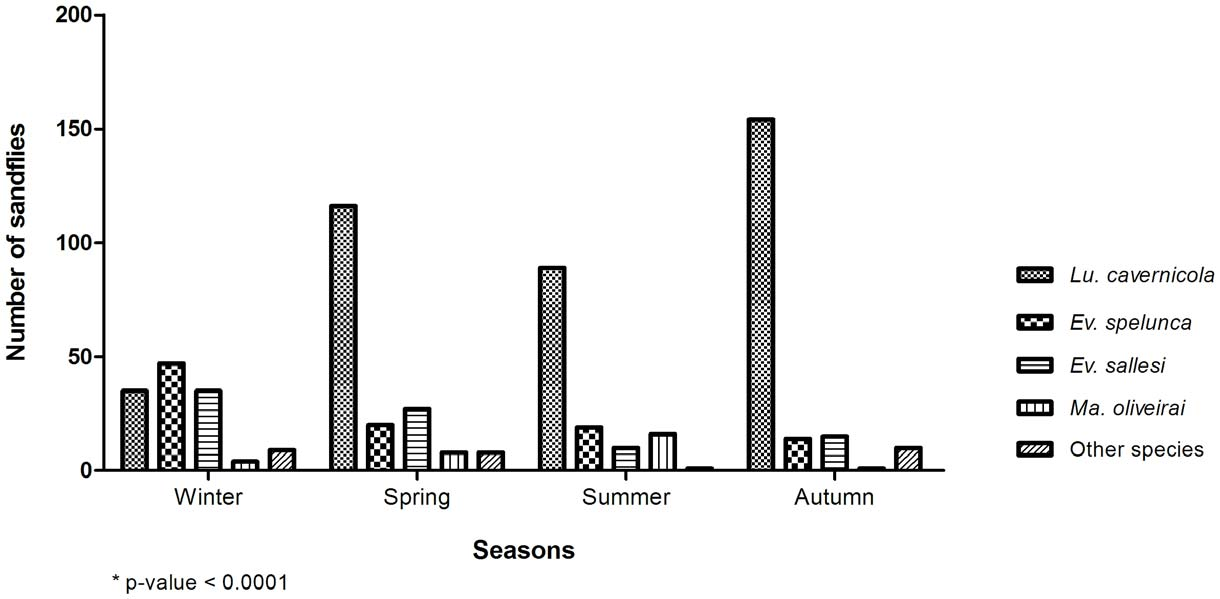
Sandfly species captured with Shannon trap in the four seasons: winter, spring, summer, autumn, inside the cave, Lassance/MG.

Data analysis in the four seasons (Figure 4) shows, however, that when considering the species caught, a statistically significant difference (p-value<0.0001) occurs.

**Figure 5:**
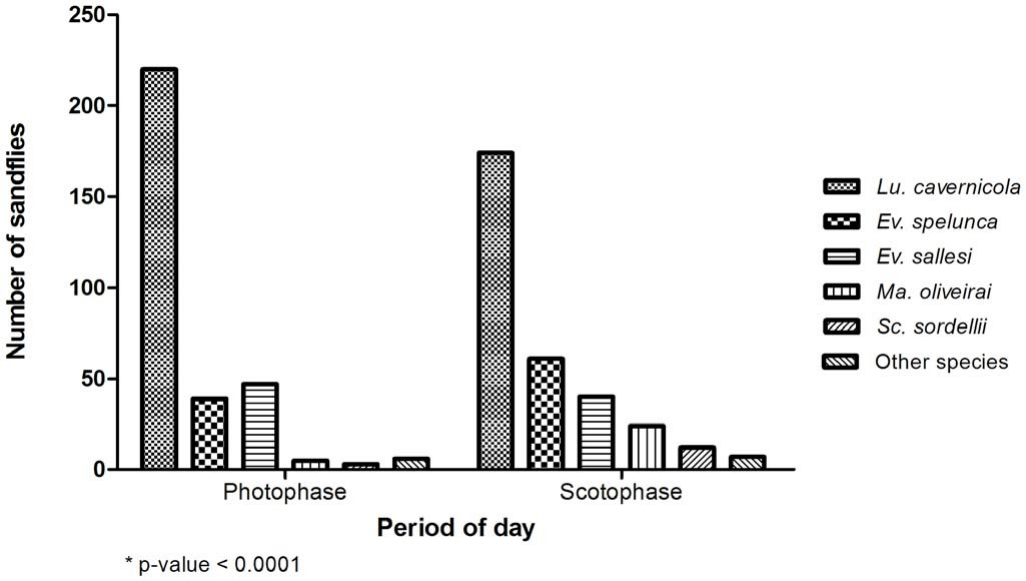
Comparison of the proportions of specimens per species of sandflies captured in winter, spring, summer and autumn, in the aphotic zone of the cave, over the LD cycle (photophase/scotophase) - Lassance/MG – Brazil.

The distribution of total sandflies captured (across all four trials) over the LD cycle was represented by 50% for each phase of the cycle. However, this distribution does not take into account the comparison per species in the two periods (scotophase/photophase) and in this case this same distribution shows a statistically significant difference (chi-square test: p-value<0.0001) (Figure 5). Thus, the purpose of this analysis was to verify whether the faunal composition varied between the two periods (photophase/scotophase). Figure 5 draws attention to the most abundant species (*Lu. cavernicola*, *Ev. spelunca* and *Ev. sallesi*) during photophase, and greater amounts of the first and last species were captured during this phase.

**Figure 6:**
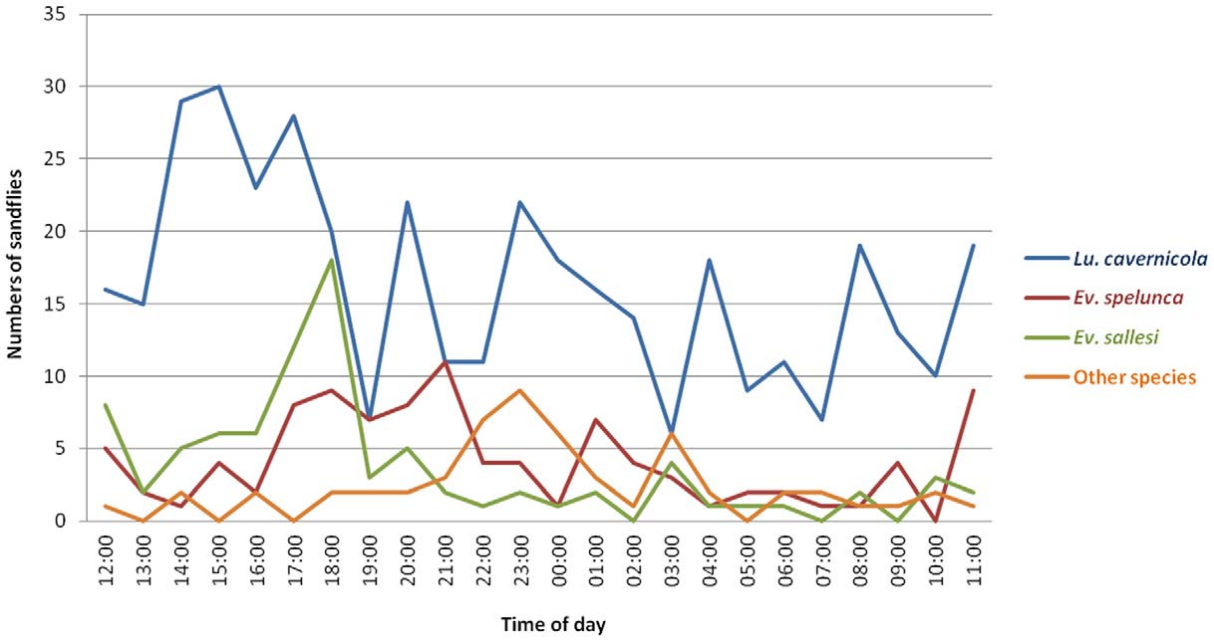
Hourly activity of the most abundant species captured with Shannon trap in winter, spring, summer and autumn, in the aphotic zone of the cave - Lassance/MG – Brazil.

Compared to the most abundant species, with emphasis over its distribution across the same 24 hours of collection, one can note that they were all captured during the entire LD cycle, which is, recorded in virtually all hours of capture. *Lu. cavernicola* was undoubtedly, the species that showed the most regular distribution throughout the 24 hours (Figure 6).

### Natural infection research

Surprisingly in a single capture, conducted in August 2010, five specimens were found through the technique of dissection to be naturally infected with flagellated forms: two *Ev. spelunca*, two *Ev. sallesi* and one *Sciopemyia sordellii* (Shannon & Del Pont, 1927). A suspension containing PBS 1× with antibiotics (streptomycin and penicillin) and intestinal contents of insects was inoculated in hamsters and in culture medium NNN-Schneider. No growth of protozoa in culture was found, and all of them were contaminated with bacteria and/or fungi that could have prevented cultures form becoming established. No clinical signs were observed in hamsters with inoculated flagellate suspensions. We examined the presence of *Leishmania* DNA in sacrificed hamsters using ITS1-PCR [14], however, when using this technique we were unable to identify the parasites found. The slides with infected specimens were photographed and filmed and after the isolation procedures they were stained.

## Discussion

Caves are considered stable environments when compared to epigean habitats and are also characterized by a permanent lack of light far from entrances [3], [15]. Fauna adapted to these conditions can be classified according to their level of adaptation: troglobites (animals that present unique modifications to cave environments), troglophiles (adapted animals, but devoid of modifications that can also develop in the external environment) and trogloxenes (animals that use caves for shelter or refuge) [16].

Although caves are apparently inhospitable environments for sandflies, some studies have shown that caves may harbor sandflies [7], [17], [18], [19]. This shows the importance of these breeding sites for such insects. Most sandflies have trogloxenos habits, almost always using caves as a refuge or shelter. However, little is known about the behaviour of sandflies in cave environments. Recently, a new species of sandfly, *Lutzomyia maruaga* (Alves, Freitas & Barret 2008) was found in a cave in the Amazon region, which shows that it is at least facultatively parthenogenetic and autogenic, as previously observed for *Lutzomyia mamedei* by Brazil and Oliveira [20], and therefore regarded as a kind of troglobite. Other studies have also described new species of sandflies reported from cave environments [8], [9]. *Evandromyia spelunca* is a new phlebotomine species of the *cortelezzii* complex described from this cave [9]. This species might show evidence of adaptation to the cave environment since it was captured in this study, in all months, was the third most abundant species and demonstrated a consistent distribution over the LD cycle.

This work has shown that in fact the phlebotomine fauna of the cave environment may present some peculiarities in regards to their behaviour. Of the total 11 species captured in this study, all of them can also be found in the external environment (unpublished data), agreeing with other findings where the diversity and density of sandflies captured in caves can be equal to or greater than surrounding environments [21], [22].

Regarding sandfly period of activity, there are few evaluative studies on this behaviour. Such studies focus on species of vectoral importance in different environments, but none in cave environment. One of these studies has addressed the behaviour of *Nyssomyia intermedia* (Lutz & Neiva, 1912) and *Nyssomyia neivai* (Pinto, 1926), which are species incriminated in the transmission of cutaneous leishmaniasis in the peridomestic area of Iporanga, state of São Paulo, where 70% of the specimens of both species were collected between 18:00 and 24:00 [23]. In another study the activity period of sandflies, also *Nyssomyia neivai*, in the endemic zone of cutaneous leishmaniasis, in Tucuman, Argentina, also showed that the hourly activity of this specie was mainly nocturnal [24].

Most man-biters feed at dusk and during the evening but *Psychodopygus wellcomei* (Fraiha, Shaw and Lainson, 1971), *Psychodopygus carrerai* (Barreto, 1946), and others will attempt to feed in the daytime as well. Windless or nearly windless conditions, along with other optimal conditions, may suddenly induce greater-than-expected numbers of sandflies to seek their hosts. Bloodfeeding females may also release aggregation pheromones that attract other females to the host [25].

Unlike almost always observed in studies of sandfly activity outside the cave environment, where a general habit of these insects is crepuscular/nocturnal, our study revealed that the cave environment presents a fauna with different behaviour. The results found by the sum of four catches for 24 hours, show 50% of the sandflies captured inside the cave, during the photophase (6:00–17:00 ), a finding that deserves attention because it differs from most reports of the behaviour of these insects.

Through graph 3, it was important to observe that in the winter and spring the peaks of occurrence of sandflies occur after dusk (during the twilight), contrary to what was observed in the summer, when the peaks were observed more frequently at night (20:00 to 0:00) and in the fall when the peaks were started in the early afternoon and ended in the early evening. However, we do not have enough data to infer hypotheses about these differences found. But, on all four catches, sandflies were found in the period of photophase.

These data led us to examine whether all species caught inside the cave had the same distribution over 24 hours. From this analysis we found that the most abundant species, mainly *Lu. cavernicola*, were responsible for the large number of individuals captured during the photophase. This brings us to the hypothesis that these species mayhave a trogloxen behaviour, but in a way that is suited to this type of environment, maybe presenting a circadian rhythm different from that observed for other species of sandflies.

This result draws attention to the vectoral capacity of these insects, coupled with the existence of vertebrates, also trogloxens, inside the caves, increasing the chances of a parasite transmission cycle occurring in this type of environment and the chances of visitors being infected if bitten by sandflies occasionally in daylight hours. These species probably have altered their biological behaviour in this environment, becoming more opportunistic and increasing their chances of transmission in this biotope.

The finding of flagellated forms in five sandflies, namely: two *Ev. spelunca*, two *Ev. sallesi* and one *Sc. sordellii*, draws attention to a possible cycle inside the cave at the time of capture. In a previous study also conducted in Lassance [26], promastigotes were found in the pyloric region and in the abdominal midgut of *Ny. neivai* and *Ev. sallesi*. Insects found by microscopic examination were macerated in saline solution and inoculated into hamsters. Subsequent analysis by polymerase chain reaction-restriction fragment length polymorphism revealed both isolates to belong to the species *Leishmania infantum chagasi*.

All samples showed no trace of blood in the digestive contents, thus increasing the actual possibility that the parasite had developed in the digestive tract of these species. However, the techniques used for the isolation of parasites were not successful in their identification and characterization. The stained forms will be submitted to other methodologies in an attempt to identify the flagellates.

The report of species incriminated in the transmission of leishmaniasis, such as *Migonemyia migonei* (França, 1920), *Nyssomyia whitmani* (Antunes & Coutinho, 1939), *Ny. intermedia* and *Lutzomyia longipalpis* (Lutz & Neiva, 1912), should be taken into consideration. Although the findings of natural infection have not permitted the identification of the *Leishmania* sp. parasite, the presence of these species inside the cave can be crucial for the existence of a cycle in this environment. Though found in small percentages in these catches, these species showed that they may occasionally visit the cave environment and opportunely could come to disseminate the parasites to hosts/reservoirs that are also present this habit.

Further studies on the biological behaviour and natural infection of sandflies caught in caves are important to the development of new hypotheses or to the finding of new cycles of disease in this ecotope, which is an environment with significant ecological and biological aspects and that has relevant unique characteristics. Furthermore, our findings have shown the relevance of studies that will account for the assessment of this type of environment, since there is a high demand for it for many different types of activities.
